# An Analysis of Italian Nurses' Approach to Patients' Pain: A Nationwide Online Survey

**DOI:** 10.1155/2018/5165262

**Published:** 2018-04-23

**Authors:** Chiara Angeletti, Cristiana Guetti, Martina Paesani, Silvia Colavincenzo, Alessandra Ciccozzi, Paolo Matteo Angeletti

**Affiliations:** ^1^Operative Unit of Anesthesiology, Intensive Care and Pain Medicine, Civil Hospital G. Mazzini of Teramo, Teramo, Italy; ^2^Struttura Operativa Dipartimentale Complessa, Cure Intensive per il Trauma e Supporti Extracorporei, Azienda Ospedaliero-Universitaria Careggi, Firenze, Italy; ^3^Department of Life, Health and Environmental Sciences, University of L'Aquila, L'Aquila, Italy

## Abstract

Healthcare providers play a fundamental role in evaluating pain. Several issues about how nurses are educated remain unsolved. The aim of our study was to address how Italian nurses manage patients suffering from pain in daily practice. A cross-sectional survey was administered among Italian registered nurses. Data were collected using a 34-item questionnaire that had been previously validated during a pilot study. The lowest level of participation/education/information events was observed in the South (*p*=0.0001). A significant difference among the four areas was found in the department affiliation of responders (*p*=0.0001). Pain assessment at patients' admission was most frequent in the Northeast (32.9%), whereas the lowest frequency was found in the South (15.1%) (*p*=0.0001). The prevalence of nurses' knowledge of pain scales and their distribution in usual applications was similar in the Northwest and -east, and Central Italy, but lower in the South (*p*=0.0001). This study underlines the need for change in the clinical approach to pain treatment in healthcare. Pain assessment is a fundamental step for preparing individualized therapeutic plans, and nurses play a crucial role in improving the quality of life of suffering patients.

## 1. Introduction

Pain is a neurophysiological phenomenon that has affected humans forever; in the last decades, social awareness about its management has improved. In clinical practice, healthcare professionals must deal with the requests of patients in our hypertechnological medical systems.

The prevalence of chronic pain was found to be between 12% in Spain and 30% in Norway [1]. The prevalence recorded for Italy was 26%. At the country level, the percentage range of severe pain carriers ranged from 32% in the UK to 50% in Israel; for Italy, this value was 43% [1]. Pain prevalence was found to be higher in northern regions (32%) than in southern regions (22%) [2].

van Hecke et al. [3] found that chronic noncancer pain affects 20% of the European population and is more frequent in women, the elderly population, and people with sociodemographic, clinical, psychological, and biological risk factors. A recent literature review conducted by Reid et al. [4] showed that the one-month prevalence of chronic noncancer pain of moderate to severe severity can be estimated at 19%.

Pain, as a symptom associated with other pathological conditions, has a prevalence ranging from 40% to 63% in hospitalized patients [5, 6] peaking at 82.3% in oncological patients in advanced or terminal stages of disease [2]. The prevalence of pain in elderly patients not living in institutions ranges from 25% to 50% and from 45 to 80% in those living in elderly care facilities [7].

An Italian study [8] reported an estimated prevalence of chronic pain (more than three months) equal to 21.7% of the entire Italian population (approximately 13 million people). Of these, 41% said they had not received adequate pain control, indicating that in Italy the care response to people in pain is still poor. Pain is undertreated in cancer patients in about 25% of cases; this prevalence may peak up at 64% in some subgroups. In patients who had finished curative treatment, the prevalence of pain was 33% (95% CI 21–46); patients who were being treated with an anticancer therapy experienced a prevalence of 59% (95% CI 44–73); patients characterized by advanced/metastatic or terminal disease experienced a prevalence of 64% (95% CI 58–69); and the majority of patients (at all stages of disease) experienced a prevalence of 53% (95% CI 43–63). In conclusion, the pooled prevalence of pain was >50% for all types of cancer [3].

A fundamental aspect of modern healthcare is the prevention of advanced oncological diseases: many European countries have solid cancer prevention campaigns [9–12]. Elderly residents in health facilities are at highest risk for inadequate pain treatment [7]. Among European countries, Italy ranks first in the clinical use of nonsteroidal anti-inflammatory drugs (NSAIDs) and at the bottom for opiate use [13]. Despite the existence of evidence-based guidelines for the appropriate management of pain, many patients still suffer from inadequate pain treatment [14, 15]. Cultural barriers negatively affect the management of pain [14]; these barriers include the educational training of healthcare professionals, including physicians and nurses, and correct patient information [16]. Healthcare providers also play a fundamental role in the initial evaluation of pain, the short-term control of pain, the analgesic effect of treatment, long-term follow-up, and the satisfaction of patients suffering from pain [17]. Many studies have shown that nurses having inadequate knowledge of pain management may negatively affect the outcomes of suffering patients [16–18]. Several issues regarding the education of nurses remain unsolved; these aspects have been dealt with in Italian law number 38 of March 2010, entitled “Instructions for the access to palliative care and pain therapy.”

The aim of this study was to understand how Italian nurses manage patients suffering from pain in daily practice and pain evaluation practices in hospital departments in different regions of the country to investigate the reception of Law 10 and to assess nurses' educational needs.

## 2. Methods

The study was a cross-sectional survey administered online from October 2013 to September 2014 to Italian registered nurses to investigate their approach to pain management. Of the 422,875 nurses in the country, 696 from all regions of Italy took part in the survey. A total of 193 males with a mean age 40.16 ± 9.5 years (mean ± standard deviation) and 503 females with a mean age 41.69 ± 9.3 years (ages ranged from 22 to 63 years for both genders) were included. The sample size was estimated using the following parameters: sample error *E*=0.04, event occurrence proportion *p*=0.5 (in case of maximum variability), and probability 1 − *α*=0.95.

### 2.1. Data Collection

Data were collected online using a questionnaire drawn by the following scientific societies: the National Federation of Nurses Colleges (IPASVI) and the Italian Association for Pain Study (AISD). The survey was anonymous, included 34 multiple choice items, and had been previously validated during a pilot study. The members of IPASVI and AISD were invited to participate in the survey via dedicated web pages of their own scientific society web sites (http://www.ipasvi.it, http://www.aisd.it, and http://www.painnursing.it). The survey was promoted by major social networks (Facebook, Twitter, and LinkedIn). The questionnaire included the following sections: (i) demographic data (gender, age, and city), (ii) professional data, department, and qualification (pediatric nurse or head nurse); (iii) participation in training courses focused on pain management, type of course (meetings, symposia, and online courses), and degree of validity/efficacy and appreciation of these courses; (iv) registration and quantification of pain (e.g., pain as the fifth vital sign, knowledge of scales for pain evaluation, frequency of these scales' application and impact on clinical decisions, and the role of nurses in the registration of pain symptoms); and (v) knowledge of Italian laws about pain. Italian regions participating in the study were grouped into four geographical areas according to the Italian National Institute of Statistics (ISTAT): the Northwest, the Northeast, Central Italy, South, and the Islands. Two groups of work departments were defined according to clinical and assistance characteristics: Group 1 included emergency/urgent care departments/wards, general and cardiologic intensive care units, oncology and hematology units, hospice/home-care, and pain treatment division; Group 2 included the remaining surgical and medical departments (pediatric, internal medicine, neurology, obstetrics, and gynecology).

### 2.2. Statistical Analysis

Data were analysed by grouping patients in four geographical areas: the Northwest, the Northeast, Central Italy, and the South. The *χ*^2^ test was used to estimate the association between categorical variables being studied. A *t*-test was used to evaluate continuous variables. Wilcoxon signed-rank and Kruskal–Wallis tests were applied to interval and ordinal variables. A value of *p* < 0.05 was considered statistically significant. SAS software was used for the statistical analyses.

## 3. Results

### 3.1. General Data on the Survey's Responders

The distribution areas of participants are summarized in Table 1. No age differences were found among the areas (the Northwest, the Northeast, Central Italy, and South Italy) (*p*=0.41). A significant gender difference was noted (*p*=0.004): Of the 665 people who were interviewed (95.5%), 503 were females (72.3%), as expected in the Italian gender distribution of nurses. An important statistically significant difference between the four areas was found for department affiliation responders (*p* < 0.0001). With reference to Group 1, the greatest concentration of participants was in the South (58.5%), while the lowest was observed in the Northeast (30.5%). Regarding occupation, our results showed that pediatric nurses were the least represented among the responders compared to nurses and head nurse (*p*=0.03).

### 3.2. Education

A statistically significant difference was detected concerning education (Table 1). In particular, the lowest level of participation in education/information events was observed in the South, whilst the highest level of education was found in the Northeast (*p* ≤ 0.0001) (i.e., these events were deemed most useful in the Northeast (*p*=0.002)).

### 3.3. Pain Evaluation

Table 2 presents data relating to pain evaluation in the hospital facilities of the four geographic areas. Pain assessment at admission was most frequent in the Northeast (32.9%) and least common in the South (15.1%) (*p*=0.0001). Prevalence of nurses' knowledge of pain scales was similar in the Northwest (95.9%), the Northeast (95.3%), and Central Italy (92.7%), but lower in the South (69.2%) (*p*=0.0001). A similar distribution in the usual application of pain evaluation scales was found: the Northwest (81.0%), the Northeast (84.5%), Central Italy (72.6%), and the South (37.1%) (*p*=0.0001). The most commonly used pain scale was VAS, followed by V-NRS, with a range of 85.2% in the Central region to 77.27% in the South and 18.7% in the Northeast and 7.2% in the South, respectively. The structured questionnaires revealed that instruments such as the McGill Pain Questionnaire/BPI and qualitative scales were poorly applied in clinical situations. Treatment plans including scales of pain assessment were also less frequent in the South (75.5%) compared to the remaining three areas (*p*=0.0009). Knowledge of devices and invasive procedures for pain treatment had the following frequency distribution: the Northwest (51.5%), the Northeast (55.4%), Central Italy (38.7%), and the South (42.1%) (*p*=0.0007). The simultaneous presence of a physician and a nurse as referring persons for pain management had the highest frequency in Central Italy (51.6%) compared to the remaining areas (*p* < 0.0001). Thereafter, variables were stratified according to the referring department (Table 3). As expected, Group 1 (emergency/urgent care departments, intensive care, cardiology intensive care, oncology/oncohaematology, and hospice/home care units) had the highest score for dedicated staff compared to other departments or units (*p*=0.0001), as well as for knowledge of devices and invasive procedures for pain treatment (59.3%) (*p*=0.0001).

### 3.4. Pain as the Fifth Vital Sign and Law Number 38

A statistically significant difference has been revealed regarding the consideration of pain as the fifth vital sign, with the following frequency distribution: the Northwest (88.1%), the Northeast (81.2%), Central Italy (70.1%), and the South (69.1%) (*p* < 0.0001). It is relevant that the highest percentages of nurses working in places where pain is not considered a vital parameter were found in Central Italy (29.9%) and in the South (30.8%). Nurses from the South were less aware of the existence of a law in Italian legislation which makes the evaluation of pain mandatory (61.4%) (*p*=0.0001).

Globally, the pain problem is considered sufficiently treated and taken into consideration by nurses following this trend in the different areas evaluated: the Northwest, 57.4%; the Northeast, 60.1%; Central Italy, 58.1%; and the South, 63.5%. A statistically significant difference for this issue was found in the department group responders (*p*=0.001).

## 4. Discussion

Pain relief is a fundamental right; nurses, as healthcare providers, have a central role in this context [13, 19–22]. From triage in an emergency department to postoperative care, from home care to palliative care at the end of life, the crucial professional figure is the nurse, who today requires specific knowledge of how to manage pain. The nurse enters fully into the overall care of the patient experiencing pain. Nurses represent an essential component in the patient pain management team: in all healthcare scenarios, they often represent the only daily caregivers who are continually expected to practice symptom management. Nurses spend more time with patients and are able to assess and manage patient pain effectively. They also play a key role in the initial assessment, control, and follow-up of analgesic treatment. However, numerous surveys conducted over the last few years, both nationally and internationally, have shown that nursing staff often lack sufficient knowledge of how to manage pain and the specific skills to treat it [23–28]. Inadequate knowledge and attitudes of nurses with regard to pain management significantly worsen the outcome of suffering patients [28]. The identification of nodes to be solved in terms of the definition of the role of the nurse in pain and the adjustment of standards of care must be a priority for those working in any healthcare environment where the pain is prevalent or even present. These issues, which remain unresolved today, were addressed in Italy by law number 38, “Provisions to ensure access to palliative care and pain therapy,” promulgated in March 2010.

### 4.1. Education in and Knowledge of Pain Management

Education of nurses and care providers should be seen as a strategic method in order to create an adequate culture of and attract attention to the problem of pain [29]. The present analysis has identified major differences in education, particularly related to the geographic area of the study sample. A higher level of qualification and commitment in nurses' education was present in the Northeast, while a lower commitment was identified in the South of Italy. From this analysis, it appears that differences in the institutional and financial commitment for the persistent education and professional improvement of nurses represent a major issue. In fact, the differences found in the four geographical areas in which ISTAT divides the national territory reflects the diffusion of specific pain therapies and palliative care services, which, according to latest report by the Ministry of Health on the implementation of law number 38, are more common in Northern and Central Italy [30]. Recent studies report that the majority of nurses involved in pain management admit to lacking adequate knowledge and instruments to address this challenge [31, 32]. As reviewed by Mattacola et al. [33], a limited number of studies are available regarding nurses' knowledge of and attitudes toward pain management in Italy compared to other European, North American, or emerging countries [24–28, 34, 35]. Nurses' education in pain management is badly needed due to the increased awareness on this issue, according to Italian law number 38 and the international community [36]. The existence and diffusion of dedicated services could be a driving force for continuing training and research in pain therapy. However, training in the treatment of pain and the role of the nurse in symptom management should start early in the course of study. A recent review by Chow and Chan [32] shows not only how the knowledge of pain, and of the problems associated with it, is scarce among nursing students but also how it can be optimal after proper training [32]. Not only primary education but also continuous education should be seen as an important investment, because knowledge is not usually automatically transferred in daily assistance; unfortunately, two different attitudes toward this problem have been identified in Italy. Basic knowledge, its application, and attention to the needs of suffering patients are issues of major importance. Basic knowledge and practical expertise alone, however, are not sufficient to change nursing practice unless basic principles of pain evaluation and management in daily practice are concomitantly standardized [37]. Several reports in the literature have analysed the importance of education and demonstrated that education may change nurses' attitudes and improve their knowledge and professional behaviour [28, 38–40]. After attending educational events, indeed, nurses become fully aware of methods of managing pain [38, 41]. This was also a relevant aspect of our survey, and one which reflected a high degree of satisfaction and utility of educational events about pain, although the percentage of nurses attending these courses was very heterogeneous across the country (Table 1).

### 4.2. Pain Evaluation

This study indicates that nurses from the North (-east and -west) correctly use the pain rating scales both at the first physical examination and later on as they register a patient's parameters during the entire time span of assistance, in order to provide reference measurements for planning care and treating pain itself. A negative attitude is still present in a high percentage of nurses operating in the South of Italy, where pain is evaluated only after patients have insistently complained and required care. At this point, pain must be promptly relieved; this misguided attitude causes discomfort for the patient and interrupts the daily activities of nurses, who are often operating in crowded hospital wards with undersized staffs, are underpaid, and burdened with excessive duties. This national situation corresponds with that reported in the literature. A few studies examined the degree of application of a rating scale (numeric, nominal, analogical, or illustrated) [42]; available data from the literature indicate that this scale is used in about 50% of cases [22, 27, 28, 36, 37]. At the national level, a gap in the use of this scale was observed between the northern (95%) and southern regions (69%). Some reports indicated that nurses use a simple interview or nonstandardized methods, or even omit the evaluation [40] in the belief that this duty belongs to the physician rather than to them [35, 42–44]. Pain evaluation is often based on reports obtained by the patient or on alleged levels of pain in patients unable to communicate [25, 43]. As in other international settings, nurses mostly complain about the lack of time for pain evaluation due to assistance duties, particularly during exhausting work shifts [26, 43, 44]. These problems have been widely documented in our survey. Pain should be quantified with a numeric parameter: this crucial aspect of assisting patients has been underlined by law number 38. Pain assessment allows for treatments to be standardized. The analysis of data from South Italy indicates that little attention is paid to pain evaluation at the time a patient is admitted, thereby limiting the subsequent approach to the patient. A direct consequence of this attitude is the poor quality of care perceived by patients and the low level of analgesia achieved. Considering hospital departments and wards, the geographical differences among nurses were less evident. Among nurses working in emergency or surgical departments, oncology hospices, or community care, expertise regarding and knowledge of technical devices and protocols were similar in the different Italian regions.

Our study indicates that nurses from the North (-east and -west) fully understood the rule of law number 38. This North-South gradient was also evident in the results of our survey; in fact, nurses from the Northern and Central Italy showed greater awareness of patients' pain than those in the South. Nurses in the Southern Italy said they were not aware of the existence of a law that requires the measurement of pain as the fifth vital sign, which also affects their familiarity with the instruments used to measure pain. The definition of professional reference figures may guarantee adequate attention to the problem of pain.

Adequate management of pain in healthcare settings may indeed result in shorter hospitalizations, fewer complications and comorbidities, fewer drugs being administered during the rehabilitation phase, fewer analgesic side effects, reduced fear related to opioid use, and an overall shorter time and less expensive rehabilitation. Our investigation indicates that there is no homogeneous treatment of patients with pain from the nursing point of view across the different areas of the country. The nursing staff in many situations does not seem to be in a position to assess pain both in terms of time and internal organization of the departments and in terms of tools and skills to better address the problem of pain management. Emerging problems for nurses seem mainly to concern inadequate knowledge, limited possibilities to assess and manage pain, and finally a reluctance to use pain assessment tools and to consider pain a vital parameter. Nurses must be aware of their central role and responsibility and must be informed about their profession. Appropriate training and continuous updating will enable healthcare personnel to achieve the necessary level of expertise in pain assessment and management needed to bridge the gap between our country and others in Europe and around the world. The most urgent problem to solve is to clearly define the border between the role of nursing and medical competence, a limit that has been overcome in other countries but which is still rather confused in Italy.

## 5. Limitations

The responders to the questionnaire may not be a representative of the Italian nurse population. Indeed, it was a relatively small group of nurses who were strongly motivated or simply were aware of the existence of the survey and were given the opportunity to participate; a possible limitation is therefore that the questionnaire was not submitted to a group of nurses selected according to a specific rule or work setting, and thus the distribution of the responders in the groups analysed could be subject to unknown biases. Drawing any definitive conclusion on differences between geographical areas is also difficult. New cross-sectional studies are needed to investigate the full application of the law, the role of nurses in the regional and national management of pain, and the usefulness of training in this field.

## 6. Conclusion

The present study aimed at emphasizing the need for a change in the clinical approach to pain treatment. Pain as a disease is an emergency that must be faced with a multidisciplinary approach; in this setting, nurses around the world play a central role, as they are directly involved in the care of patients suffering from cancer- or noncancer-related pain. Pain assessment is a fundamental step for preparing individualized therapeutic plans. Thus, pain should be seen a vital parameter and assessed several times in the course of 24 hours. Nowadays, a primary duty of nurses should be to offer personalized assistance and elaborate care and take part in research in order to improve the quality of life of suffering patients.

## Figures and Tables

**Table 1:** Distribution of participant features.

Variable	Total responders		Northwest		Northeast		Central		South		*p*
	Number	%	Number	%	Number	%	Number	%	Number	%	
Gender											
Male	665	95.5	44	26.0	43	20.2	45	36.3	53	33.3	
Female			125	74.0	170	79.8	79	63.7	106	66.7	0.004
Age group											
25–35	665	95.5	27	16.0	29	13.6	26	21.0	14	8.8	
35–45			52	30.8	52	24.4	27	21.8	49	30.8	0.41
45–55			57	33.7	93	43.7	50	40.3	65	40.9	
>55			33	19.5	39	18.3	21	16.9	31	19.5	
Occupational category											
Nurse	665	95.5	136	80.4	178	83.6	111	89.5	143	89.9	
Head nurse			25	14.8	34	16.0	12	9.7	16	10.1	0.03
Pediatric nurse			8	4.7	1	0.4	1	0.8	0	0.0	
Department of affiliation											
Group 1^a^	665	95.5	55	32.5	65	30.5	40	32.3	93	58.5	
Group 2^b^			114	67.5	148	69.5	84	67.7	66	41.5	<0.0001
Participation education/information events											
Yes	665	95.5	105	62.1	140	65.7	66	53.2	66	41.5	
No			64	37.9	73	34.3	58	46.8	93	58.5	<0.0001
Utility of education/information events											
Very good	380/665	57.1	75	71.4	126	89.3	48	71.6	49	73.1	
Good			19	18.1	11	7.8	14	20.9	16	23.9	0.002
Poor			11	10.5	4	2.8	5	7.5	2	3.0	

^a^Department Group 1: surgical departments. ^b^Department Group 2: medical departments.

**Table 2:** Evaluation of pain according to geographic areas.

Variable	Total responders		Northwest		Northeast		Central		South		*p*
	Number	%	Number	%	Number	%	Number	%	Number	%	
Pain is seen as the fifth vital parameter. Is this the case in your working place?											
Yes	665	95.5	149	88.1	173	81.2	87	70.1	110	69.1	
No			20	11.8	40	18.8	37	29.9	49	30.8	<0.0001
How frequently do you assess pain in your hospital?											
When requested by the patient	664	95.4	56	33.1	65	30.5	53	42.7	97	61.4	
At patient's admission in the ward			46	27.2	57	26.8	27	21.8	26	16.5	<0.0001
Once			15	8.9	16	7.5	6	4.8	2	1.2	
Twice			51	30.2	70	32.9	31	25.0	24	15.1	
Never			1	0.6	5	2.3	7	5.7	9	5.7	
Do you know the scales for pain evaluation?											
Yes	665	95.5	162	95.9	203	95.3	115	92.7	110	69.2	
No			7	4.1	10	4.7	9	7.3	49	30.8	<0.0001
If yes, which do you know?											
VAS	590		137	84.6	161	79.3	98	85.2	85	77.27	
V-NRS			19	11.7	38	18.7	9	7.8	8	7.2	0.0002
McGill pain questionnaire			3	1.8	1	0.5	3	2.6	5	4.6	
BPI			0	0.0	0	0.0	0	0.0	1	0.9	
Qualitative scales			3	1.9	3	1.5	5	4.3	11	10.0	
Do you usually apply the scales for pain evaluation?											
Yes	665	95.5	137	81.0	180	84.5	90	72.6	59	37.1	
No			32	18.9	33	15.5	34	27.4	100	62.9	<0.0001
If no why?											
Do you recognize the complaining patient?	207		19	48.7	8	24.2	7	20.6	28	27.7	
I have no time			4	10.3	4	12.1	5	14.7	15	14.8	0.005
It is not my job			2	5.1	2	6.1	3	8.8	18	17.8	
Nobody asked me to do that			4	10.3	5	15.2	3	8.8	23	22.8	
Others			10	25.6	4	42.4	16	7.1	17	16.8	
Does the evaluation scale affect the subsequent assistance plans?											
Yes	665	95.5	149	88.2	189	88.7	110	88.7	120	75.5	
No			20	11.8	24	11.3	14	11.3	39	24.5	0.0009
Are you aware about devices or invasive procedures for pain treatment?											
Yes	665	95.5	87	51.5	118	55.4	48	38.7	67	42.1	
No			82	48.5	95	44.6	76	61.3	92	57.9	0.007
Does a reference person for pain management exist in your hospital ward?											
Yes, a physician	665	95.5	49	29.0	45	21.1	39	31.5	73	45.9	
Yes, a nurse			12	7.1	26	12.2	7	5.7	2	1.3	<0.0001
Yes, both physician and nurse			24	14.2	67	31.5	14	11.2	8	5.0	
No reference person is present			84	49.7	75	35.2	64	51.6	76	47.8	
Are you aware about the existence of a law prescribing as mandatory the measurement of pain?											
Yes	664	95.4	145	85.8	187	87.8	103	83.1	97	61.4	
No			24	14.2	26	2.2	21	16.9	61	38.6	<0.0001
Are, in your opinion, problems related to pain sufficiently taken into account by care providers in your working department?											
Yes	665	95.5	97	57.4	128	60.1	72	58.1	101	63.5	
No			72	42.6	85	39.9	52	41.9	58	36.5	0.69

VAS, visual analogue scale; V-NRS, verbal numeric rating scale; BPI, brief pain inventor.

**Table 3:** Evaluation of pain according to affiliation of departments considered.

Variable	Total responders		Department Group 1^a^		Department Group 2^b^		*p*
Pain is seen as the fifth vital sign. Is this true in your working place?							
Yes	696	100.0	217	81.9	325	75.4	0.045
No			48	18.1	106	24.6	
How frequently do you assess pain in your hospital?							
When requested by the patient	695	99.9	97	36.6	185	43.0	0.06
At patient's admission in the ward			63	23.8	103	24.0	
Once			17	6.4	23	5.3	
Twice			80	30.2	105	24.4	
Never			8	3.0	14	3.3	
Do you know the scales for pain evaluation?							
Yes	696	100.0	228	86.0	387	89.8	0.13
No			37	14.0	44	10.2	
Do you usually apply the scales for pain evaluation?							
Yes	696	100.0	188	70.9	299	69.4	0.66
No			77	29.1	132	30.6	
Does the evaluation scale affect the subsequent assistance plans?							
Yes	696	100.0	230	86.8	365	84.7	0.44
No			35	13.2	66	15.3	
Are you aware about devices or invasive procedures for pain treatment?							
Yes	696	100.0	157	59.3	174	40.4	<0.0001
No			108	40.7	257	59.6	
Does a reference person for pain management exist in your hospital ward?							
Yes, a physician	696	100.0	105	39.6	110	25.5	<0.0001
Yes, a nurse			12	4.5	38	8.8	
Yes, a physician and nurse			48	18.1	69	16.0	
No reference person is present			100	37.7	214	49.7	
Are you aware about the existence of a law prescribing as mandatory the measurement of pain?							
Yes	695	99.9	217	82.2	339	78.7	0.26
No			47	17.8	92	21.3	
Are, in your opinion, problems related to pain sufficiently taken into account by care providers in your working department?							
Yes	696	100.0	178	67.2	235	54.5	0.001
No			87	32.8	196	45.5	

^a^Department Group 1: surgical departments. ^b^Department Group 2: medical departments.

## Abbreviations

NSAIDs: Nonsteroidal anti-inflammatory drugs
IPASVI: National federation of nurses colleges
AISD: Italian association for pain study
ISTAT: Italian national institute of statistics
VAS: Visual analogue scale
V-NRS: Verbal numeric rating scale
BPI: Brief pain inventory.

## References

[1] Breivik H., Collett B., Ventafridda V., Cohen R., Gallacher D. (2006). Survey of chronic pain in Europe: prevalence, impact on daily life, and treatment. *European Journal of Pain*.

[2] Costantini M., Viterbori P., Flego G. (2002). Prevalence of pain in Italian hospitals: results of a regional cross-sectional survey. *Journal of Pain Symptom Management*.

[3] van Hecke O., Torrance N., Smith B. H. (2013). Chronic pain epidemiology and its clinical relevance. *British Journal of Anaesthesia*.

[4] Reid K. J., Harker J., Bala M. M. (2011). Epidemiology of chronic non-cancer pain in Europe: narrative review of prevalence, pain treatments and pain impact. *Current Medical Research and Opinion*.

[5] Melotti R. M., Samolsky-Dekel B. G., Ricchi E. (2005). Pain prevalence and predictors among inpatients in a major Italian teaching hospital. A baseline survey towards a pain free hospital. *European Journal of Pain*.

[6] Gianni W., Madaio R. A., Di Ciocco L. (2010). Prevalence of pain in elderly hospitalized patients. *Archives of Gerontology and Geriatrics*.

[7] De Conno F., Ripamonti C., Brunelli C. (2005). Opioid purchases and expenditure in nine western European countries: ‘are we killing off morphine?’. *Palliative Medicine*.

[8] Fanelli G., Gensini G., Canonico P. L. (2012). Dolore in Italia. Analisi della situazione. Proposte operative. *Recenti Progressi in Medicina*.

[9] Altobelli E., Rapacchietta L., Angeletti P. M., Barbante L., Profeta F. V., Fagnano R. (2017). Breast cancer screening programmes across the WHO European region: differences among countries based on national income level. *International Journal of Environmental Research and Public Health*.

[10] Altobelli E., Lattanzi A. (2015). Cervical carcinoma in the European Union: an update on disease burden, screening program state of activation, and coverage as of March 2014. *International Journal of Gynecologic Cancer*.

[11] Altobelli E., Lattanzi A., Paduano R., Varassi G., di Orio F. (2014). Colorectal cancer prevention in Europe: burden of disease and status of screening programs. *Preventive Medicine*.

[12] Altobelli E., D’Aloisio F., Angeletti P. M. (2016). Colorectal cancer screening in countries of European Council outside of the EU-28. *World Journal Gastroenterology*.

[13] Italian Ministry of Health (January 2016). *Rapporto al Parlamento sullo Stato di Attuazione della Legge n. 38 del 15 Marzo 2010*.

[14] Ogston-Tuck S. (2012). A silent epidemic: community nursing and effective pain management. *British Journal of Community Nursing*.

[15] Maryland Board of Nursing (January 2016). *Pain Management, Nursing Role/Core Competency, A Guide for Nurses*.

[16] Mac Lellan K. (2004). Postoperative pain: strategy for improving patient experiences. *Journal of Advanced Nursing*.

[17] Bernhofer E. (2011). Ethics: ethics and pain management in hospitalized patients. *Online Journal of Issues Nursing*.

[18] Wilson B. (2007). Nurses’ knowledge of pain. *Journal of Clinical Nursing*.

[19] Shahnazi H., Saryazdi H., Sharifirad G., Hasanzadeh A., Charkazi A., Moodi M. (2012). The survey of nurse’s knowledge and attitude toward cancer pain management: application of health belief model. *Journal of Education and Health Promotion*.

[20] Rose L., Smith O., Gélinas C. (2012). Critical care nurses’ pain assessment and management practices: a survey in Canada. *American Journal of Critical Care*.

[21] Ferrell B. (2005). Ethical perspectives on pain and suffering. *Pain Management Nursing*.

[22] Italian Ministry of Health (January 2016). *Relazione sull’Attuazione delle Disposizioni per Garantire l’Accesso alle Cure Palliative e alla Terapia del Dolore*.

[23] Lewthwaite B. J., Jabusch K. M., Wheeler B. J. (2011). Nurses’ knowledge and attitudes regarding pain management in hospitalized adults. *Journal of Continuing Education in Nursing*.

[24] Ware L. J., Bruckenthal P., Davis G. C., O’Conner-Von S. K. (2011). Factors that influence patient advocacy by pain management nurses: results of the American society for pain management nursing survey. *Pain Management Nursing*.

[25] Al-Shaer D., Hill P. D., Anderson M. A. (2011). Nurses’ knowledge and attitudes regarding pain assessment and intervention. *Medsurg Nursing*.

[26] Layman Young J., Horton F. M., Davidhizar R. (2006). Nursing attitudes and beliefs in pain assessment and management. *Journal of Advanced Nursing*.

[27] Lui L. Y., So W. K., Fong D. Y. (2008). Knowledge and attitudes regarding pain management among nurses in Hong Kong medical units. *Journal of Clinical Nursing*.

[28] Jho H. J., Kim Y., Kong K. A. (2014). Knowledge, practices, and perceived barriers regarding cancer pain management among physicians and nurses in Korea: a nationwide multicenter survey. *PLoS One*.

[29] Jarrett A., Church T., Fancher-Gonzalez K., Shackelford J., Lofton A. (2013). Nurses’ knowledge and attitudes about pain in hospitalized patients. *Clinical Nurse Specialist*.

[30] Marchand S. (January 2016). What is pain?. *The Phenomenon of Pain*.

[31] (January 2016). http://www.salute.gov.it/imgs/C_17_pubblicazioni_2360_allegato.pdf.

[32] Chow K. M., Chan J. C. Y. (2015). Pain knowledge and attitudes of nursing students: a literature review. *Nurse Education Today*.

[33] Mattacola P., Serio F., Mauro L., Fabriani L., Latina R. (January 2016). *Le Conoscenze e le Attitudini degli Infermieri nella Gestione del Dolore: una Revisione Narrativa della Letteratura*.

[34] Bernardi M., Catania G., Lambert A., Tridello G., Luzzani M. (2007). Knowledge and attitudes about cancer pain management: a national survey of Italian oncology nurses. *European Journal of Oncology Nursing*.

[35] Di Muzio M., Barbato D., Maria S. M. (2010). *Pain Management: uno Studio Infermieristico*.

[36] Office of The Army Surgeon General (January 2016). *Pain Management Task Force Report Providing a Standardized DoD and VHA Vision and Approach to Pain Management to Optimize the Care of Warriors and Their Families*.

[37] Berry P. H., Dahl J. L. (2000). The new JCAHO pain standards: implications for pain management nurses. *Pain Management Nursing*.

[38] Abdalrahim M. S., Majali S. A., Stomberg M. W., Bergbom I. (2010). The effect of postoperative pain management program on improving nurses’ knowledge and attitudes toward pain. *Nurse Education in Practice*.

[39] Michaels T. K., Hubbartt E., Carroll S. A., Hudson-Barr D. (2007). Evaluating an educational approach to improve pain assessment in hospitalized patients. *Journal of Nursing Care Quality*.

[40] Patiraki E. I., Papathanassoglou E. D., Tafas C. (2006). A randomized controlled trial of an educational intervention on Hellenic nursing staff’s knowledge and attitudes on cancer pain management. *European Journal of Oncology Nursing*.

[41] Lin P. C., Chiang H. W., Chiang T. T., Chen C. S. (2008). Pain management: evaluating the effectiveness of an educational programme for surgical nursing staff. *Journal of Clinical Nursing*.

[42] Willens J. S., DePascale C., Penny J. (2010). Role delineation study for the American society for pain management nursing. *Pain Management Nursing*.

[43] Rejeh N., Ahmadi F., Mohammadi E., Anoosheh M., Kazemnejad A. (2008). Nurses’ experiences and perceptions of influencing barriers to postoperative pain management. *International Nursing Review*.

[44] De Silva B. S., Rolls C. (2011). Attitudes, beliefs, and practices of Sri Lankan nurses toward cancer pain management: an ethnographic study. *Nursing and Health Sciences*.

